# DEET as a feeding deterrent

**DOI:** 10.1371/journal.pone.0189243

**Published:** 2017-12-14

**Authors:** WeiYu Lu, Justin K. Hwang, Fangfang Zeng, Walter S. Leal

**Affiliations:** Department of Molecular and Cellular Biology, University of California Davis, Davis, CA, United States of America; Centro de Pesquisas René Rachou, BRAZIL

## Abstract

The insect repellent *N*,*N*-diethyl-3-methylbenzamide (DEET), is a multimodal compound that acts as a spatial repellent as well as an irritant (contact repellent), thus being perceived by the insect’s olfactory and gustatory systems as an odorant and a tastant, respectively. Soon after DEET was developed, almost 6 decades ago, it was reported that it reduced mosquito feeding on blood mixed with this repellent. It is now known that the mosquito proboscis senses contact repellents with the tips (labella) of the labium, which remain in direct contact with the outer layers of the skin, while the stylets, including the feeding deterrent sensor (labrum), penetrate the skin. We designed a behavioral assay that allowed us to measure feeding deterrence without complications from contact or spatial repellency. Using the southern house mosquito, *Culex quinquefasciatus*, we demonstrate here that when DEET was mixed with blood and covered by Parafilm® layers, the mean number of landings and duration of contacts with surfaces covering blood mixed with DEET or blood plus solvent (dimethyl sulfoxide) did not differ significantly thus implying that DEET did not leak to the outer surface. The feeding times, however, were significantly different. When blood was mixed either with 0.1 or 1% DEET, female southern house mosquitoes spent significantly (P<0.0001) less time feeding than the time spent feeding on blood mixed only with the solvent. By contrast, significant differences in the mean times of feeding on blood containing 1% picaridin and blood plus solvent were significant at 5%, but not at 1% level. Like DEET, the contact repellent and insecticide, permethrin, caused a significant (P<0.0001) reduction in feeding time. We, therefore, concluded, that in this context, DEET, permethrin, and, to a lesser extent, picaridin, act as feeding deterrents.

## Introduction

Chemicals used to reduce mosquito bites are not only repellents sensu stricto, ie, compounds that cause the responder to steer away from the source, but are also excitorepellents or irritants, ie, chemicals eliciting increased locomotor activity after an insect makes contact with the source [1]. From a strict mechanistic viewpoint, these 2 groups should be named noncontact and contact disengagents, respectively [2]. From a more pragmatic perspective, the end result is the same, ie, mosquitoes are kept at bay by sensing odorants in the vapor phase (spatial repellents) and/or by detecting non-volatile tastants (contact repellents) upon direct contact with these chemicals (on a skin surface, for example) [3]. Although its complete mode of action is still a matter of considerable debate, DEET (= *N*,*N*-diethyl-3-methylbenzamide) is undoubtedly a multimodal compound [3, 4], which is perceived by both the olfactory and gustatory systems as an odorant and a tastant, respectively. Additionally, evidence in the literature suggests that DEET also acts as a feeding deterrent [5]. The pioneering findings by Bar-Zeek and Schmidt [5] that blood-feeding was prevented when samples were spiked with DEET has been overlooked most probably because of the difficulty in teasing apart feeding deterrence from contact repellency.

Mosquitoes sense the environment with their antennae, maxillary palps, proboscis, tarsi, and ovipositors. Whereas the antennae and maxillary palps are involved in the reception of odorants (eg, spatial repellents), the proboscis is involved in the reception of contact repellents and other tastants. This sophisticated “microneedle system” [6] comprises a gutter-like labium that encloses a fascicle. There are 2 lobes (labella) at the tip of the labium, and the fascicle contains 6 stylets: a pair of ridged maxillae, a pair of mandibles, a hypopharynx with its salivary canal, and a labrum that carries sense organs on its tip [7]. During feeding, the fascicle penetrates the host’s skin while the labium bends and the labella remains in direct contact with the outer layer of the skin [8]. Although it has been demonstrated that labral apical sensilla respond to phagostimulants [9, 10] and feeding deterrents [11], it remains difficult to unambiguously determine whether reduced feeding on DEET-spiked blood is mediated by “contact repellency” or “deterrence.” Indeed, Bar-Zeek and Schmidt [5] suggested that “repellency” was caused by low concentrations of DEET (then named diethyltoluamide) in the blood.

**Fig 1:**
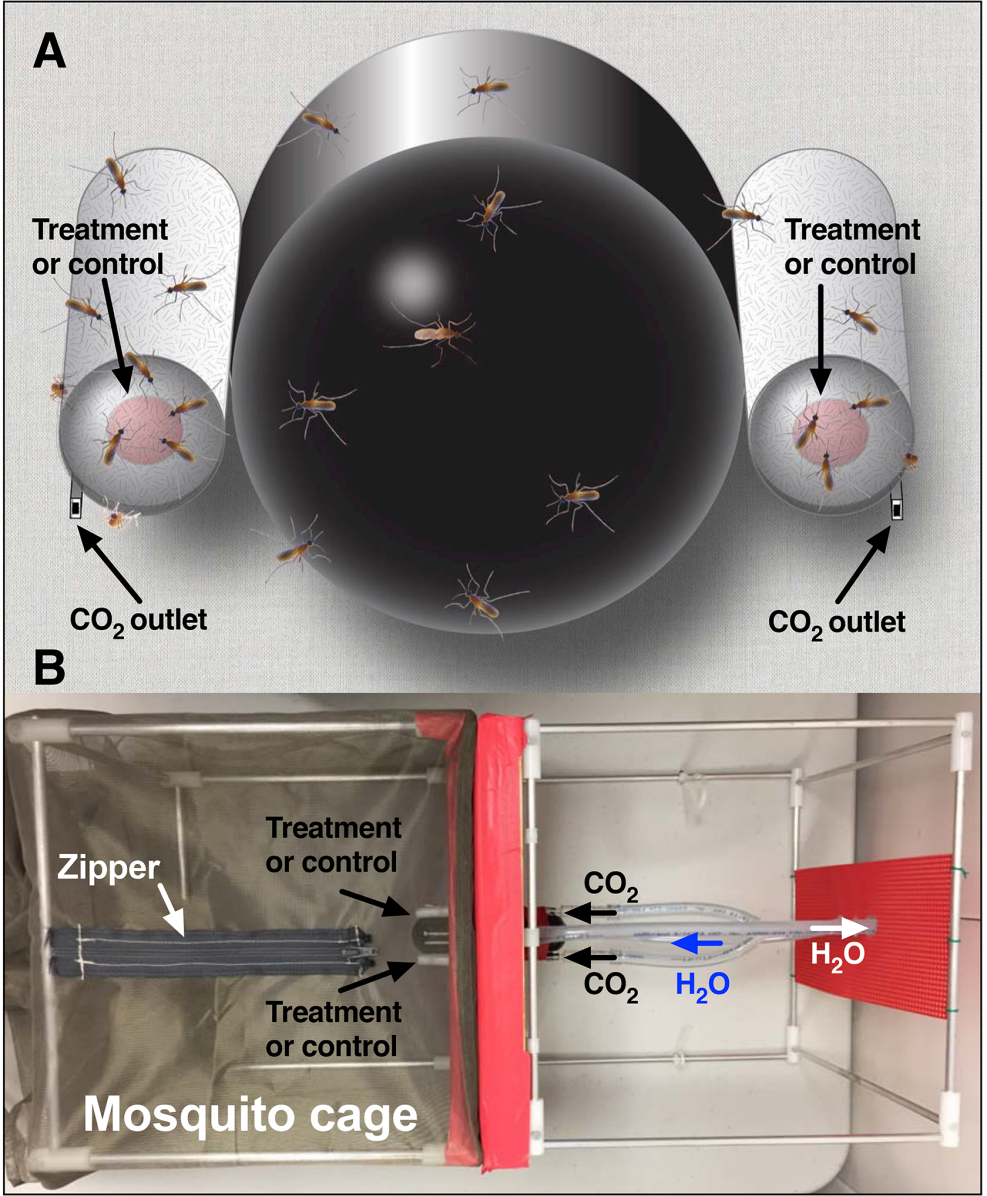
Illustration of the modified arena. (**A**) A Dudley tube painted black from inside was flanked by 2 cotton rolls secured in place by syringe needles that delivered CO_2_. Samples of defibrinated sheep blood mixed with solvent only or spiked with DEET were loaded on these cotton rolls, which were subsequently covered with Parafilm. (**B**) An aerial view of the arena. Mosquitoes were placed on a mosquito cage accessible from the top and having a camera (not shown) attached to the left. The Dudley tube was connected to a water bath (not shown) and the syringe needles to a CO_2_ tank (not shown).

To address whether reduced feeding on DEET-spiked blood was due in part to repellency or deterrence, we devised a modified version of our surface landing and feeding assay (Fig 1) [12]. We lured mosquitoes to feed on 2 cotton rolls covered with dual layers of Parafilm® sealing film and loaded with blood, one spiked with DEET and the other with solvent, and measured feeding times in the 2 parts of the arena. Here, we report that mosquitoes spend significantly less time feeding on DEET-spiked blood than on the control. Likewise, permethrin also acted as a feeding deterrent, but picaridin showed a lower response.

## Materials and methods

### Mosquitoes

*Culex quinquefasciatus* mosquitoes used in this study were originally from a laboratory colony initiated with mosquitoes collected in the 1950s in Merced, California and currently kept by Dr. Anthony Cornel (Kearney Agricultural Center, University of California-Davis). The Davis colony has been maintained separately for more than 6 years under 12:12 (L:D), 27±1°C, and 75% relative humidity.

### Behavioral arena

Feeding behavior was measured using a modified surface landing and feeding assay [12]. In brief, the device consisted of a base and a detachable assay cage (Fig 1B). The frame of the base was made from an aluminum collapsible field cage (Bioquip, 30.5 × 30.5 × 30.5 cm) with a wooden board (30 × 30 cm) attached to the front of the cage and covered with red cardstock (The Country Porch, GX-CF-1) and red lab tape. Three openings were drilled through the wooden board to accommodate one 50-mL Dudley bubbling tube (Fisherbrand, 40356) and two 16-gauge syringe needles (Sigma-Aldrich, Z108782), orientations of which are illustrated on Fig 1A. The Dudley tube painted internally with black hobby and craft enamel (Krylon, SCB-028) was attached to a water bath circulator with the temperature set at 38°C. The 2 syringe needles were connected to a CO_2_ tank through a bubbler to deliver CO_2_ at 50 mL/min. The frame of the detachable assay cage was made with the same aluminum collapsible field cage. Red cardstock was taped internally at 1 face of the cage, 1 circular opening, and 2 small holes were made in the cardstock to allow the Dudley tube and CO_2_ needles to project into the mosquito cage. The cage was completed with a field cage cover (Bioquip, 30.5 × 30.5 × 76.2 cm). One square, sealable opening (7 × 7 cm) was made at the backside of the field cage cover, allowing the Dudley tube and CO_2_ needles to insert into the cage. A slit was made on the top of the cage, and a zipper (10 cm) was sewn on to the slit for an easily accessible opening. A camera-accessible opening (d = 5 cm) with a drawstring was made at the front of the field cage (Fig 1B).

### Chemicals

DEET and permethrin (mixture of cis and trans isomers) were acquired from Sigma-Aldrich (PESTANAL^®^, analytical standards); picaridin was from a previous work [12]. Stock solutions (10% m/v) were prepared in dimethyl sulfoxide (DMSO) and diluted to 1% when needed. The blood mixtures were prepared by mixing 180 μL of defibrinated sheep blood (UCD, VetMed) with 20 μL of a 10% solution (of DEET, picaridin, or permethrin) to give a final concentration of 1%. The control was prepared in the same manner but using only DMSO.

### Behavioral measurements and statistical analysis

Fifty female mosquitoes (6 days after emergence) were aspirated and transferred to the arena 2 hours before each experiment. All openings were sealed, and the cage was kept near the base of the arena. Thirty minutes after the water started circulating, the assay cage was then inserted into the base (Fig 1). Aliquots (200 μL) of blood mixed with DMSO only or DEET in DMSO were gently pipetted onto one end of a piece of dental cotton (Primo Dental Products, #2 Medium) to make a blood circle on the cotton. A strip of Parafilm sealing film (ca. 8 x 5 cm) was stretched fully along the length and then wrapped around the cotton roll, covering the surface twice. To distinguish the treatment from the control group, a snipped insect pin (BioQuip, black enameled No.5) was tagged at the back of the cotton by a small piece of Parafilm. The sealed cotton rolls were placed in between the CO_2_ dispensing needles and the Dudley tube. Five microliters (the amount of 1 blood meal [13]) of pure defibrinated sheep blood were smeared onto the surface of the Parafilm (to prime mosquitoes to start feeding). CO_2_ flow was initiated, and the assay was recorded during the scotophase with a camcorder equipped with a Super NightShot Plus infrared system (Sony Digital Handycan, DCR-DVD 910). First, we measured the number of mosquitoes landing on both surfaces as well as the contact times to determine whether DEET would act as a spatial and/or contact repellent in this experimental set-up. Each assay lasted for 30 min. Once finished, insects were gently removed from the cotton rolls, and the assays were reinitiated with fresh sealed cotton rolls with switched positions. For each group of tested mosquitoes (each cohort of 50 mosquitoes used for 1 experiment), test and control were placed at least twice on each side of the arena to avoid possible side bias. Behavioral observations were not done in real time, but rather by retrieving the recorded videos. Mosquito-feeding duration was counted only after the blood used for priming was already dried. For measuring feeding time, we selected mosquitoes that clearly pierced the membrane by forcing its head down towards blood, stopped movement of the head and the body, and started waving the hind leg while the stylets were inserted. Once all these steps were observed, we rewound the tape and started counting the feeding time. End of feeding was determined when the proboscis was removed and mosquitoes walked away. Out of the mosquitoes that clearly pierced, we preferred mosquitoes that were feeding solitarily rather than in groups so as to avoid interruption of feeding by other mosquitoes’ interference. We limited observations to at most the first 10 mosquitoes per assay, but each experiment (with a new cage of mosquitoes) was replicated 3–9 times. Means were compared on the basis of at least 30 measurements from the control and 30 measurements from the treatment side. Treatments and their controls were compared by Mann Whitney two-tailed test using Prism 7 (GraphPad, La Jolla, CA).

## Results and discussion

### Behavioral responses

**Fig 2:**
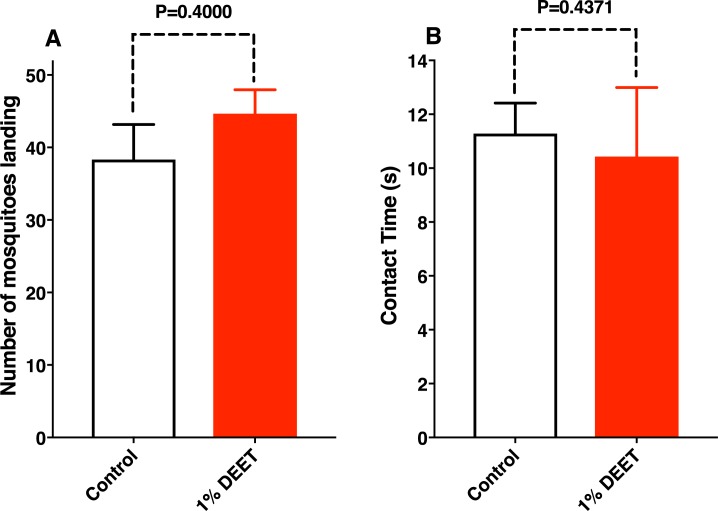
Measurements of landings and duration of contact with the surfaces prior to feeding. (**A**) The mean number of mosquitoes landing on the control and DEET sides of the arena in 15 min (first half of 30-min experiments) did not differ significantly (Mann Whitney two-tailed test, n = 3). (**B**) The contact times measured from the time the mosquitoes landed until they started feeding were not significantly different (Mann Whitney two-tailed test, n = 7).

Upon retrieving the videos, it became clear that it is highly unlikely that contact repellency was involved. Indeed, the mean number of landings on the treatment side of the arena did not differ significantly (P<0.05) from the mean number of landings on the control side (Fig 2A). Additionally, the mean time that mosquitoes spent on the Parafilm-covered blood spiked with DEET (contact time) did not differ significantly (P<0.05) from the mean time spent on the surface covering blood devoid of DEET (Fig 2B). Of note, this “residence time” on the Parafilm surfaces was recorded from the time mosquitoes landed and before feeding was initiated. As far as contact is concerned, mosquitoes behaved similarly when landing on the surfaces covering blood spiked with DEET or loaded with blood plus solvent. These observations suggest that DEET did not leak from the blood to the outer surface of the paraffin film. Therefore, the feeding times we measured next were not influenced by repellency upon contact with the surfaces. We observed that mosquitoes probed similarly on both sides of the arena; the difference in behavior was observed once they had initiated a blood meal (S1 Video).

**Fig 3:**
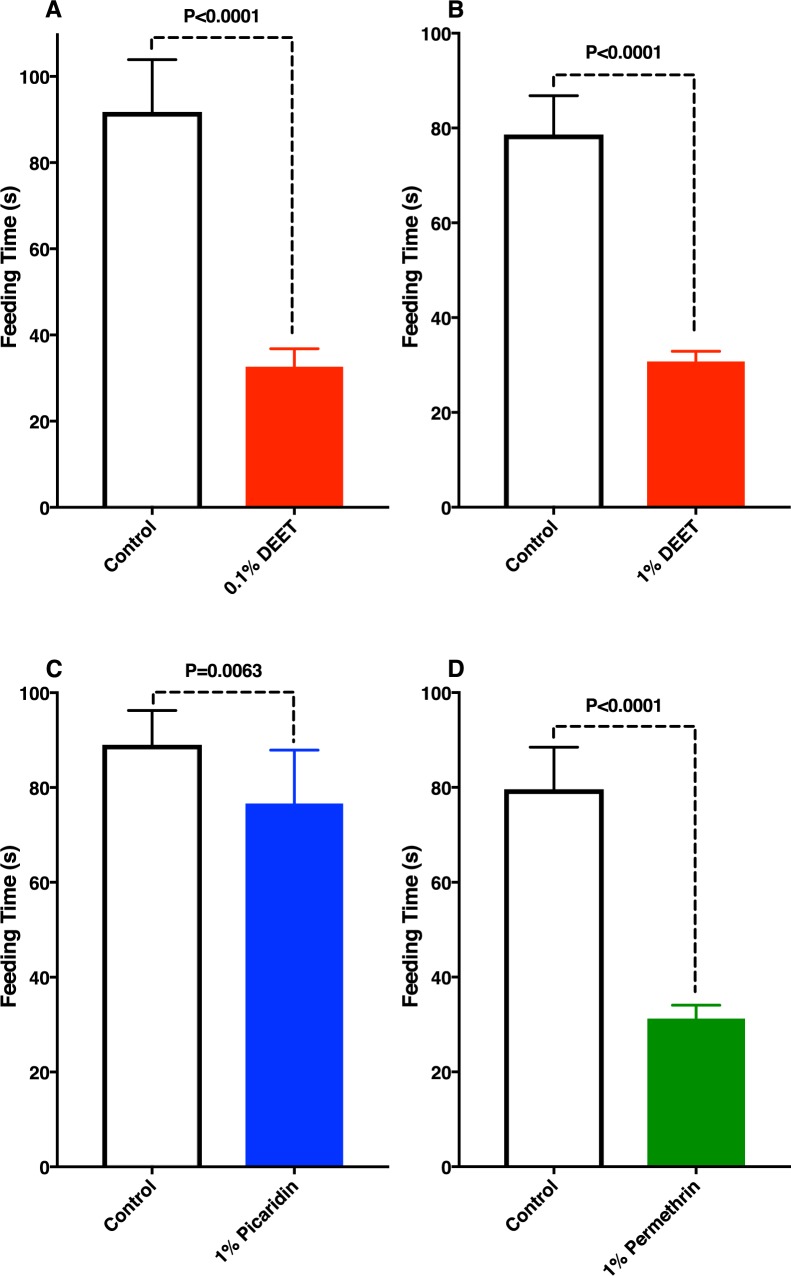
Comparative feeding times on blood mixed with solvent or test repellents. (**A**) 0.1% DEET, (**B)** 1% DEET, (**C**) 1% picaridin, and (**D**) 1% permethrin.

Mosquitoes spent significantly more time feeding on the control side of the arena than on cotton rolls loaded with 0.1% DEET-spiked blood (control, 91.8±12.1 s; DEET, 32.7±4.2 s, n = 30 measurements from 3 experiments and 4 replicates; P<0.0001) (Fig 3A). Likewise, they spent significantly less time feeding on 1% DEET-spiked blood than on blood with solvent only (control, 78.6±8.2 s; DEET, 30.8±2.1 s; n = 90 measurements from 6 experiments and 9 replicates; P<0.0001) (Fig 3B). The difference in the time feeding on blood spiked with 1% picaridin was significantly higher than in control at 5%, but not at 1% level (control, 89.0±7.2 s; picaridin, 76.6±11.2 s; n = 60 measurements from 4 experiments and 7 replicates; P = 0.0063) (Fig 3C). Although all samples were freshly prepared and tested, we cannot rule out the possibility that picaridin degraded more rapidly upon being mixed with blood.

It has been demonstrated that a DEET-sensitive odorant receptor from the southern house mosquito, CquiOR136, [14] is also expressed in the tip of the labrum [8]. Therefore, we initially surmised that mosquitoes detected DEET in the blood samples by activating this receptor. The fact that this receptor is more sensitive to picaridin than DEET coupled with the marginal response elicited by picaridin does not support this assumption. It is, therefore, likely that mosquitoes detect DEET in the blood with their gustatory system. Next, we tested the effect of permethrin, a compound commonly used in long-lasting insecticidal nets [15] given its dual property as an insecticide and excitorepellent [16]. Of note, permethrin is not a ligand for CquiOR136 [14]. Like DEET, permethrin had a significant deterrent effect, with mosquitoes feeding significantly less on permethrin-spiked blood than on blood containing only DMSO (control, 79.6±8.8 s, permethrin, 21.8±2.8 s; n = 60 measurements from 5 experiments and 6 replicates; P<0.0001) (Fig 3D).

## Conclusions

With a modified version of the surface landing and feeding assay [12], we were able to demonstrate that reduced feeding on blood spiked with DEET was due to a deterrent rather than contact repellency effect. In this experimental setup, we provided blood on cotton rolls, which were covered with 2 layers of Parafilm. DEET did not leak and, consequently, contact repellency was not at play. This is demonstrated by the fact that mosquitoes landed randomly on the various surfaces of the arena (S1 Video) and that the number and duration of the landings on the surface covering blood spiked with DEET did not differ from the similar data recorded for the side covering blood with solvent only (Fig 2). Upon direct contact of the stylets with blood, mosquitoes prematurely terminated feeding on blood spiked with DEET and permethrin, but not with picaridin. Our findings suggest that the earlier observation of “repellency” by the presence of DEET [5] in blood is due in part or fully to “feeding deterrence.” In addition to being a spatial and a contact repellent, DEET is also a feeding deterrent. Previously, it has been suggested that DEET is a feeding deterrent due to contacts with treated surfaces [17]. By contrast, our findings show that feeding is deterred by direct contact with a blood meal. Whereas the 2 well-known properties of DEET are essential for reducing mosquito bites and, consequently, transmission of diseases, “feeding deterrence” is of less importance in medical entomology given that once mosquitoes are already in contact with the blood they may have already transmitted arbovirus.

## Supporting information

S1 Video Illustration of DEET-elicited feeding deterrence. (MP4)

S1 Dataset Raw data for Fig 2. (CSV)

S2 Dataset Raw data for Fig 3. (CSV)
